# Leptin Enhances Availability of Apoptotic Cell-Derived Self-Antigen in Systemic Lupus Erythematosus

**DOI:** 10.1371/journal.pone.0112826

**Published:** 2014-11-17

**Authors:** Gil Amarilyo, Noriko Iikuni, Aijing Liu, Giuseppe Matarese, Antonio La Cava

**Affiliations:** 1 University of California Los Angeles, California, Los Angeles, United States of America; 2 Università di Salerno, Baronissi, Italy; 3 IRCCS MultiMedica, Milan, Italy; INSERM-Université Paris-Sud, France

## Abstract

In systemic lupus erythematosus (SLE), the availability of self-antigen promotes and fuels self-reactive immune responses. Apoptotic cells represent a major source of self-antigens, and an impairment of the removal of apoptotic material containing self-antigen can contribute to the development of autoimmunity. To address whether the adipocytokine leptin - which favors autoimmune responses through little understood mechanisms - could modulate the handling of apoptotic cells in SLE, we evaluated the ability of leptin to modulate the capacity of macrophages to phagocytose apoptotic bodies in (NZB×NZW)F_1_ lupus mice. It was found that leptin promoted phagocytosis of apoptotic cells by macrophages by modulating cAMP levels in macrophages. This finding associated with an increased availability of antigen that favored the development of T cell responses to apoptotic-derived antigen. As leptin promotes macrophage phagocytosis of apoptotic bodies in SLE and subsequent availability of apoptotic-derived antigen to T cells, an inhibition of this process via leptin blockade might have a therapeutic potential in SLE.

## Introduction

Apoptotic cell death and subsequent clearance of apoptotic bodies have an important role in the maintenance of immune tolerance. A dysregulation of apoptosis and/or an altered clearance of apoptotic material can lead to the development of autoimmunity. [1] For example, a defective clearance of apoptotic cell-derived material has been described in systemic lupus erythematosus (SLE) patients and in lupus-prone mice [2]–[3].

SLE is a systemic autoimmune disease in which autoreactive immune responses attack multiple tissues, causing inflammation and tissue damage that can lead to a significant loss of organ function. The reasons why the course of SLE can alternate periods of illness with remissions are not well understood, although there is consensus that those aspects may be driven by factors that modulate the inflammatory state of the patient. [4] As a consequence of this gap of information, the treatment of SLE mainly relies on the use of immunosuppressive drugs that inhibit immune responses in a rather non-specific manner, to reduce inflammation [5].

Leptin is an adipocytokine that regulates metabolism [6] and modulates immune responses. [7] Leptin has structural similarities with the cytokines of the long-chain helical family, and displays proinflammatory properties that are most evident in inflammation and in autoimmunity. [7]–[8] The levels of this adipocytokine are increased in infection and in inflammation, or after exposure to inflammatory stimuli such as LPS, TNF-α, and IL-1. [7] Circulating leptin is also elevated in most SLE patients, and appears to contribute to proinflammatory events associated with the disease [9]–[10].

Since the generation and availability of self-antigen are important elements in the development and maintenance of tissue inflammation in SLE, we investigated how leptin influenced those aspects in the pathogenesis of SLE. In particular, we focused on the fact that the clearance of apoptotic cells by macrophages is critical for the control of tissue homeostasis, and that an impairment in the uptake of apoptotic cells can lead to the generation of autoantibodies to nuclear antigens and the activation of (auto)immune cells. [11]–[12] We found that leptin promoted availability of apoptotic cell-derived antigen in lupus mice which, in turn, favored expansion of antigen-reactive T cells.

## Materials and Methods

### Mice

(NZB×NZW) F_1_ (NZB/W), C57Bl6 (B6), DO.11.10 transgenic mice (that carry a TCR specific for OVA_323–339_ peptide), and leptin-receptor-deficient (*db/db*) (B6*^db/db^*) mice were purchased from The Jackson Laboratory (Bar Harbor, ME) and housed at the University of California Los Angeles (UCLA). The study was carried out in strict accordance with the recommendations in the Guide for the Care and Use of Laboratory Animals of the National Institutes of Health with a protocol approved by the UCLA Animal Research Committee. Female mice, aged 8–10 weeks, were used in the experiments.

### Reagents

Recombinant mouse leptin was purchased from R&D Systems (Minneapolis, MN). Anti-leptin Ab was from Cell Sciences (Canton, MA). Before use, reagents were treated with Pierce High Capacity Endotoxin Removal Resin (Thermo Scientific, Rockford, IL).

### Flow cytometry

Phenotypic analyses were performed with combinations of fluorochrome-conjugated Ab (all from eBioscience, San Diego, CA) using standard techniques and acquired with a FACSCalibur flow cytometer (BD Biosciences, San Jose, CA). Data analysis was done using FloJo software (Tree Star Inc., Ashland, OR).

### Cell preparation

Jurkat T cells purchased from the American Type Culture Collection (ATCC, Manassas, VA) were labeled with CFSE (carboxyfluorescein succinimidyl ester; Sigma-Aldrich, St. Louis, MO) and made apoptotic (as confirmed in flow cytometry by annexin V and 7-aminoactinomycin (7-AAD) staining using BD Biosciences kit) following exposure for 10 min at 254 nm UV and subsequent incubation for 2 h in medium. Necrotic cell death was induced by incubation at 55°C for 15 min. [13] Each of these treatments consistently resulted in >85% cell death, as determined by changes in forward and side scatter characteristics in flow cytometry (not shown).

### Phagocytosis *in vivo*

To recruit macrophages into the peritoneum, mice were injected i.p. with 3 ml of 3% thioglycolate. Although thioglycolate treatment associated with a ∼10-fold increase in the number of circulating macrophages, it did not influence their surface expression of leptin receptor (assessed by flow cytometry as MFI, not shown). Contralateral i.p. injection of recombinant mouse leptin (6 consecutive doses of 2 µg/g at 12-h intervals) or vehicle was given starting at the time of thioglycolate treatment. At day 3 post-injection, mice were infused i.p. with 1×10^7^ CFSE-labeled apoptotic Jurkat cells. After 30 min, peritoneal cells were recovered. To remove erythrocytes and non-phagocytosed bodies, cells were incubated on polystyrene dishes for 1 h, and then washed. For determination of macrophage uptake of apoptotic cells (CFSE^+^) by flow cytometry, collected cells were stained with PE-conjugated anti-mouse CD11b Ab. For confocal microscopy, cells were resuspended in 1% cold BSA/PBS, centrifuged in a Shandon Cytospin 3 centrifuge (Thermo Fisher Scientific, Waltham, MA), and dried sediments were fixed with −20°C acetone, blocked with 3% BSA/PBS, and stained with anti-mouse CD11b Ab before visualization with a LSM 310 laser scanning confocal microscope (Carl Zeiss Inc., Toronto, Canada).

### Phagocytosis *in vitro*

Three days after thioglycolate injection, peritoneal macrophages were collected from abdominal cavities and co-cultured in serum-free HL-1 medium (Lonza, Anaheim, CA) with CFSE-labeled apoptotic Jurkat T cells (1×10^7^ cells) in the presence of scalar doses of leptin. After 2 h, cells were stained with anti-mouse CD11b Ab (BioLegend, San Diego, CA). Macrophage uptake of apoptotic cells was assessed by flow cytometry as co-staining for CFSE and anti-CD11b Ab.

### Measurement of cAMP

cAMP levels were detected using the Cyclic AMP EIA Kit (Cayman Chemical Co., Ann Arbor, MI), according to the manufacturer’s instructions.

### Antigen presentation assay

After 1 h adherence of peritoneal macrophages at 37°C in HL-1 medium, non-adherent cells were removed by washing, and macrophages cultured for 2 h at 37°C with 20 µg/ml ovalbumin (OVA), labeled with TAMRA (5-carboxytetramethylrhodamine; Genaxxon Bioscience, Ulm, Germany). Adherent cells were then washed, sorted for TAMRA dye positivity, and induced to apoptose by treatment with 1 µg/ml lipopolysaccharide (LPS) (Sigma-Aldrich) for 4 h followed by addition of 5 mM ATP (Sigma-Aldrich) for another 45 min. Harvested material was washed before use as source of OVA-containing apoptotic bodies.

To study OVA-TCR transgenic T cell responses to OVA-containing apoptotic bodies, OVA-TCR transgenic DO11.10 mice were immunized s.c. with 2 µg OVA (Sigma-Aldrich) emulsified 1∶1 in complete Freund adjuvant (CFA) (Sigma-Aldrich). After 72 h, mice were sacrificed, splenocytes stained with CFSE, and cocultured with TAMRA-labeled apoptotic bodies containing OVA (see above) in the presence of scalar doses of leptin or vehicle for 48 h before staining with clonotype-specific KJ1.26 Ab (eBioscience) and flow cytometry for enumeration of OVA-specific T cells.

### Statistical analyses

Two-sided *t* test was used for two-group comparisons. Statistical analyses were done using Prism 5 software (GraphPad, San Diego). A p-value <0.05 was considered significant.

## Results and Discussion

### Leptin promotes lupus macrophage phagocytosis *in vitro*

**Figure 1:**
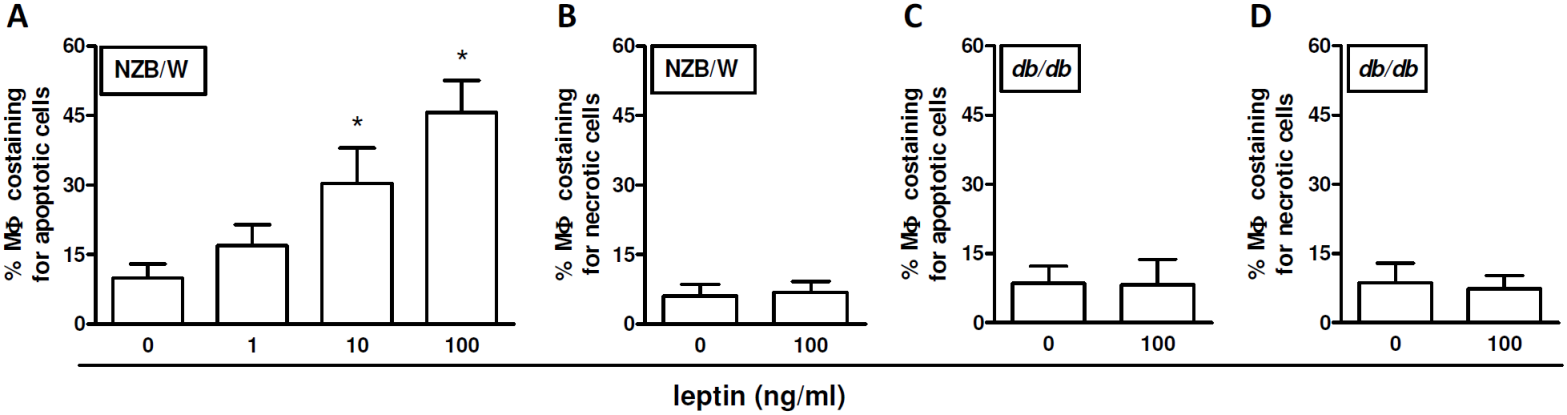
Leptin modulates the uptake of apoptotic cells *in vitro* from lupus macrophage. Peritoneal macrophages (MΦ) from NZB/W lupus mice (A–B) or leptin-receptor-deficient (non-autoimmune) *db/db* mice (C–D) were co-cultured *ex vivo* with 1×10^7^ CFSE-labeled apoptotic cells (A, C) or necrotic cells (B, D) in the presence of scalar doses of leptin (*x* axis). After 2 h, cells were stained with PE-labeled anti-mouse CD11b Ab and the labeled phagocytosed material within macrophages (PE-positive) was visualized as co-staining in flow cytometry. Additional details are reported in the Methods. *p<0.01 vs no (0) leptin.

In SLE, the impaired clearance of apoptotic cells causes an accumulation of cellular material that contains self-antigen. [1]–[2], [14] To test a possible role of leptin in this process, we co-cultured - in the presence of scalar doses of leptin - purified macrophages derived from NZB/W lupus mice (obtained according to the protocol shown in figure S1) together with CFSE-labeled apoptotic cells. After 2 h in co-culture, a dose-dependent increase in the uptake of apoptotic cells by lupus macrophages was observed in the presence of increasing concentrations of leptin (figure 1A). Leptin did not modulate the phagocytic capacity of macrophages for non-apoptotic cells (figure 1B). Specificity of the results was indicated by lack of effects of leptin on macrophages in leptin-deficient (*db/db*) macrophages (figure 1C–D).

### Leptin promotes lupus macrophage phagocytosis *in vivo*

**Figure 2:**
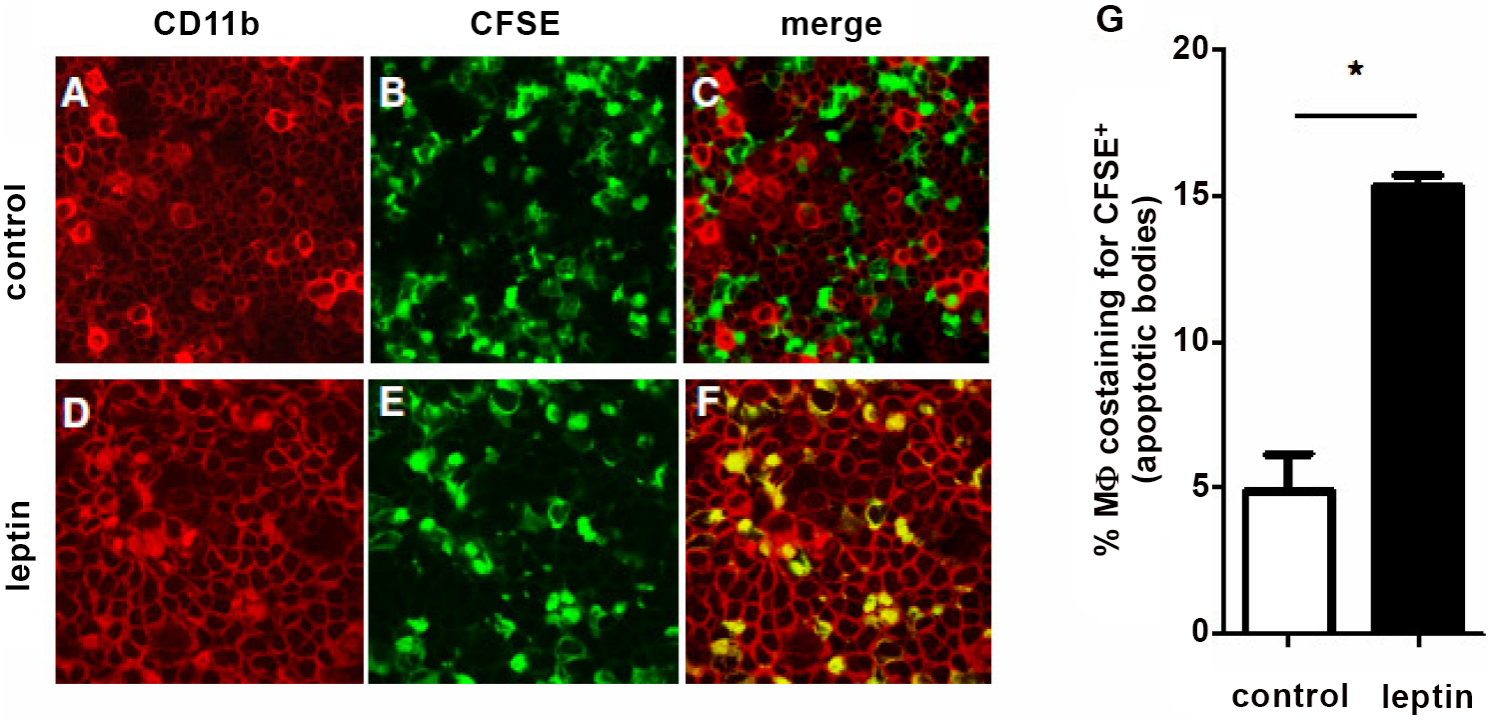
Leptin promotes phagocytosis by lupus macrophages *in vivo*. NZB/W mice were injected i.p. with thioglycolate prior to injection of either vehicle (A–C) or 2 µg/g leptin (D–F) at 12-h intervals. After 72 h, 1×10^6^ CFSE-positive apoptotic cells were injected i.p. After 30 min, uptake of apoptotic cells by CD11b^+^ macrophages (PE) in peritoneal fluid of recipient animals was visualized *ex vivo* by confocal microscopy as colocalization of PE-positive macrophages (red) and CFSE-positive (green) apoptotic bodies within the same cell (yellow). Representative of three experiments (n = 6 per group). Original magnification: 10x. (G) Cumulative flow cytometry of peritoneal macrophages costaining for apoptotic cells. *p<0.01 vs control.

**Figure 3:**
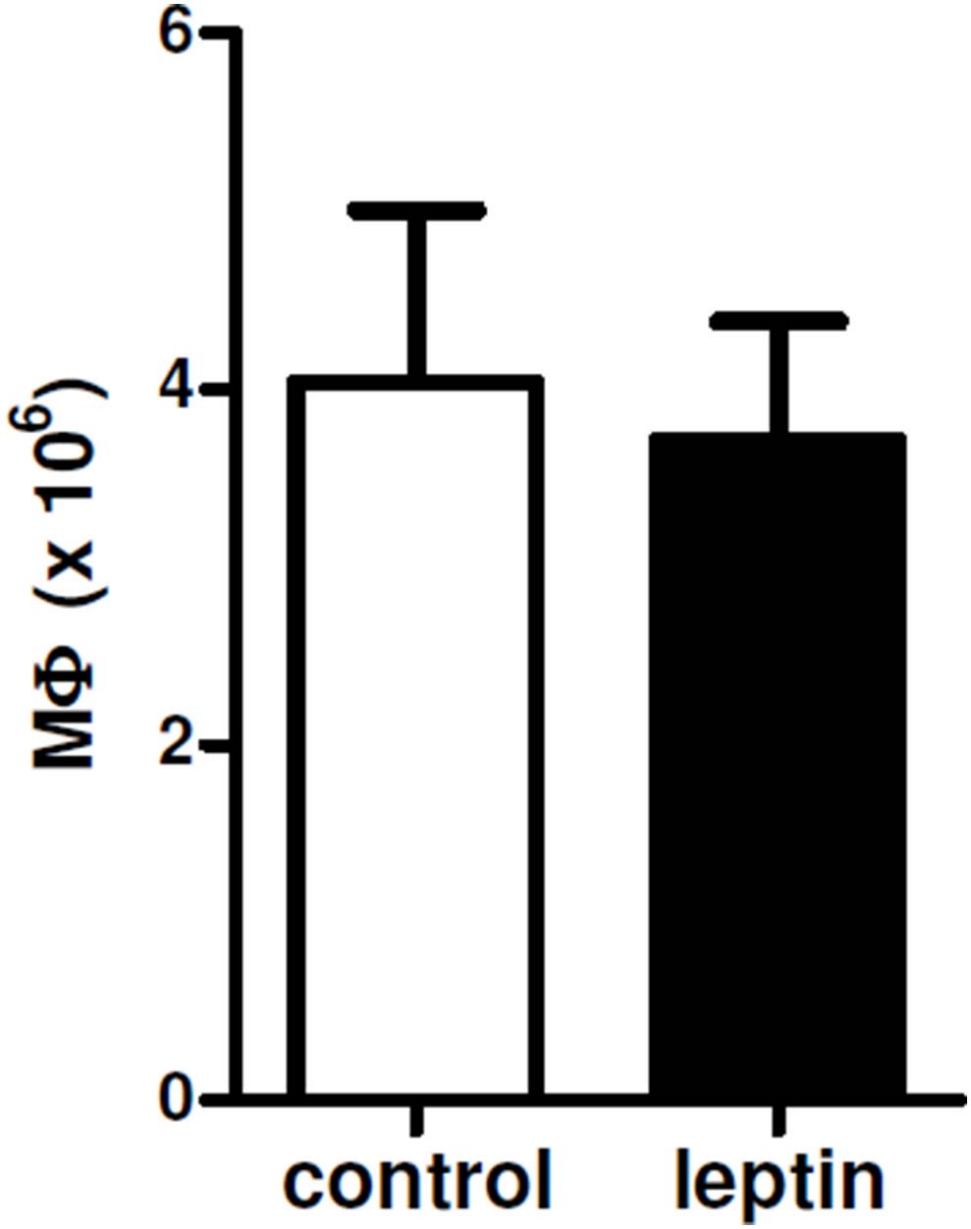
Leptin does not influence peritoneal macrophage recruitment. Total number of peritoneal macrophages recovered three days after i.p. thioglycolate in NZB/W mice in that had been injected i.p. with vehicle (control) or leptin. Further details can be found in the Methods. p not significant.

Since the above findings suggested an ability of leptin to promote phagocytosis in macrophages, we determined whether similar effects could also be observed *in vivo*. To this aim, labeled apoptotic cells were injected i.p. into two groups of NZB/W mice that had been pre-treated with thioglycolate 3 days earlier. One group of mice received leptin in addition to apoptotic cells, the other (control) group of mice received vehicle together with apoptotic cells. It was found that leptin-treated mice had a significant increase in macrophage phagocytosis of apoptotic cells when compared to control mice that had been treated with vehicle (figure 2A–F). In particular, leptin-treated mice displayed a ∼3-fold increase in the frequency of phagocytosed apoptotic cells as compared to controls (figure 2G). Those effects of leptin on macrophages were lupus-specific as not observed in control C57Bl.6 mice under the same experimental conditions (not shown) and were not due to a modulation of macrophages recruitment *in vivo*, because the total numbers of peritoneal macrophages were comparable between animals treated with leptin or with vehicle (figure 3).

### Leptin modulates cAMP levels in macrophages

cAMP is produced during phagocytosis [15], where it acts as second messenger in a signaling cascade that leads to downstream activation of the suppression of phagocytosis and facilitation of the production of proinflammatory mediators [16].

**Figure 4:**
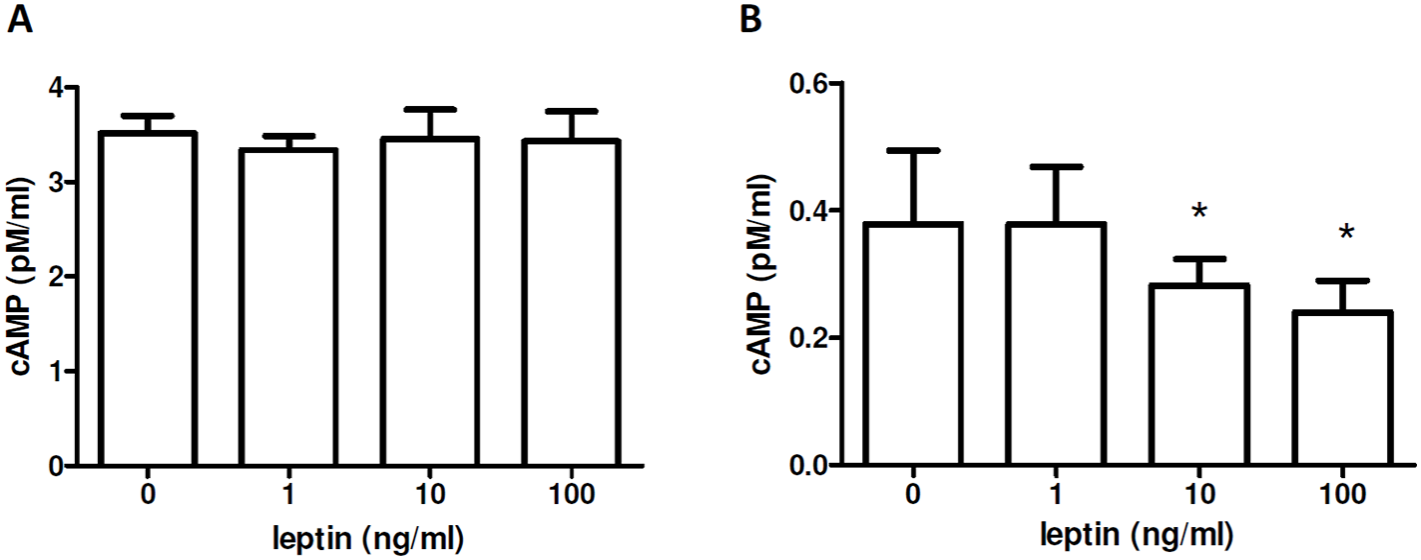
Effects of leptin on cAMP levels in macrophages. cAMP was measured as intracellular (A) and in supernatant (B) of NZB/W macrophages cultured 1 h with scalar doses of leptin (see Methods for further details). Representative of three experiments in triplicate. *p<0.05 vs control (0)

The analysis of the levels of cAMP in macrophages cultured in the presence of scalar doses of leptin showed no significant changes in intracellular cAMP expression (figure 4A). However, cAMP levels were reduced in supernatants of cultured macrophages in the presence of increasing concentrations of leptin (figure 4B), suggesting a transient effect of leptin on macrophage production of cAMP that is not maintained over time.

### The uptake of apoptotic cells induced by leptin in macrophages promotes proliferation of T cells reactive to apoptotic-cell antigen

**Figure 5:**
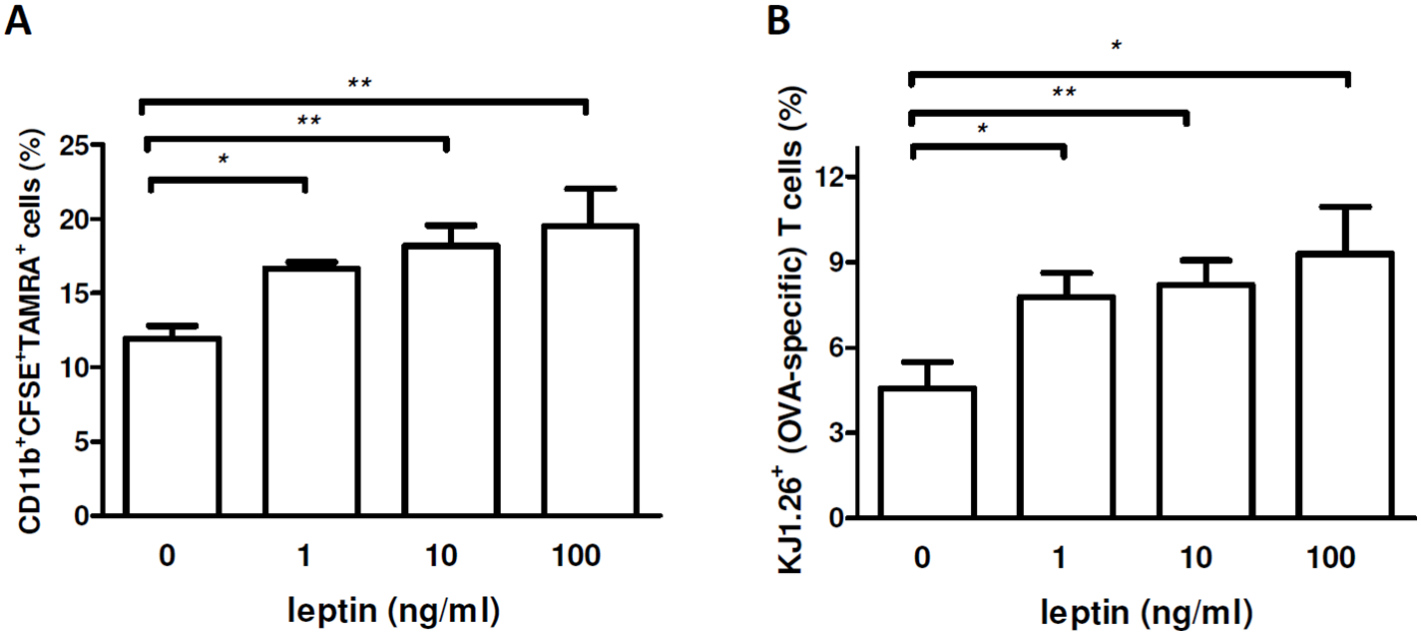
Increased macrophage uptake of apoptotic cells induced by leptin promotes proliferation of self antigen-reactive T cells. TAMRA-labeled apoptotic bodies containing OVA (see Methods) were co-cultured with CFSE-labeled macrophages for 2 h, followed by staining with APC-labeled anti-CD11b Ab and flow cytometry. (A) Frequency of TAMRA^+^CFSE^+^ CD11b^+^ cells (macrophages positive for OVA-containing apoptotic bodies) in OVA-TCR transgenic mice (DO11.10) after culture in the presence of scalar doses of leptin. (B) Flow cytometry staining with clonotype-specific KJ1.26 Ab (OVA-specific TCR) 48 h after co-incubation of OVA-immunized DO11.10 (OVA-TCR transgenic) mouse splenocytes with macrophages containing OVA-loaded apoptotic bodies (TAMRA^+^). *p<0.05, **p<0.01 vs control (n = 5).

Macrophages that phagocytose apoptotic cells contain the self-antigen that is present in apoptotic bodies. [17] Self-antigens are known to represent driving factors in the generation and maintenance of autoimmune responses. [18] Considering that our results had indicated an ability of leptin to facilitate the uptake of apoptotic cells by macrophages (figures 1 and 2), we assessed the impact of availability of intracellular (apoptotic-derived) antigen to macrophages on the proliferation of T cells reactive to that antigen. Macrophages from DO11.10 mice (carrying the DO11.10 transgenic TCR reactive to OVA) were fed OVA, stained with TAMRA, and made apoptotic (see Methods for more details). Apoptotic bodies containing OVA (TAMRA^+^) were then incubated with CFSE-labeled splenocytes from OVA-immunized DO11.10 mice for 2 h in the presence of scalar doses of leptin. Flow cytometry for CD11b^+^TAMRA^+^CFSE^+^ cells showed that leptin had promoted phagocytosis of OVA-containing apoptotic bodies in the splenic macrophages (figure 5A). Additionally, the increased frequency of DO11.10 clonotype-specific KJ1.26^+^ T cells reactive to OVA 48 h after treatment with leptin in the cocultures of splenocytes with OVA-loaded macrophages indicated a promoting activity of leptin on the proliferation of the splenic OVA-reactive T cells (figure 5B). This finding is consistent with our recent observations that showed that leptin could promote survival and proliferation of autoreactive CD4^+^ T cells [19]–[20].

Since leptin levels are abnormally elevated in NZB/W lupus mice and in SLE patients, [9]–[10] these results suggest the possibility that leptin could sustain autoimmune responses by facilitating availability of apoptotic material that contains antigen that can be recognized by antigen-specific T cells. In this sense, our findings extend mechanistically the prior work that showed an enhanced phagocytosis in murine macrophages induced by leptin [21], where (type 1) macrophages (M1) expressing the leptin receptor responded to leptin, producing pro-inflammatory IL-6 and TNF-α [22].

In summary, this manuscript shows that leptin can promote, via cAMP, the phagocytosis of apoptotic bodies and subsequent proliferation of T cells reactive to apoptosis-derived antigen. These results imply that limiting the above processes, e.g. via leptin blockade, could have effects in the modulation of autoimmune responses in SLE.

## Supporting Information

Figure S1 **Experimental protocol.** Mice were injected i.p. with 3 ml 3% thioglycolate for i.p. recruitment of macrophages. Treated mice were then divided into two groups: one receiving six injections of 2 µg/g of leptin per body weight at 12-h intervals, the other group receiving vehicle at the same time points. After 72 h, peritoneal macrophages were recovered for experimental use.
(DOC)
