# Supplementary material for: Leptin Enhances Availability of Apoptotic Cell-Derived Self-Antigen in Systemic Lupus Erythematosus
Source: PLoS One. 2014 Nov 17;9(11):e112826. doi: 10.1371/journal.pone.0112826 (PMC4234630; doi:10.1371/journal.pone.0112826)
Supplement: Figure S1 — Experimental protocol. Mice were injected i.p. with 3 ml 3% thioglycolate for i.p. recruitment of macrophages. Treated mice were then divided into two groups: one receiving six injections of 2 µg/g of leptin per body weight at 12-h intervals, the other group receiving vehicle at the same time points. After 72 h, peritoneal macrophages were recovered for experimental use. (DOC) [file pone.0112826.s001.doc]

Thioglycolate (i.p.)

**3 days**

Culture with labeled apoptotic cells (i.p.)

+ leptin

*Ex vivo* experiments (flow cytometry)

*In vitro* experiments (cell cultures)
